# Beyond the patient: understanding partners' sexual challenges and professional support needs during the breast cancer trajectory

**DOI:** 10.3389/fpsyg.2026.1813874

**Published:** 2026-05-21

**Authors:** Lara Vesentini, Welche Goossens, Dorien Macquoi, Marian Vanhoeij, Roel Van Overmeire, Johan Bilsen

**Affiliations:** 1 Mental Health and Wellbeing Research Group (MENT), Vrije Universiteit Brussel, Brussels, Belgium; 2 Breast Clinic, University Hospital Brussels, Brussels, Belgium

**Keywords:** breast neoplasms, communication, psychosocial support, qualitative research, sexuality, spouses

## Abstract

**Background:**

Partners of women with breast cancer face several challenges, often affecting their sexual wellbeing. Because their specific needs remain underexplored, this exploratory study aims to get a deeper understanding of partners' sexual experiences, as well as their needs for professional support across different stages of the breast cancer trajectory.

**Methods:**

In-depth interviews were conducted with four partners of women with breast cancer and analyzed thematically in Belgium.

**Results:**

Breast cancer affected partners emotionally, such as feeling powerless, and leaded to taking on additional responsibilities. They reported to feel underrecognized and to experience decreased sexual desire and activity, partly due to the body alterations of the women. Communication was difficult for some couples. Information about sexuality and professional support for partners is currently lacking. Contrary to the dyadic coping approach, most partners preferred to receive professional support individually.

**Conclusions:**

Providing partners with individually tailored information and professional support seems to be beneficial.

## Introduction

Breast cancer is the most prevalent cancer among women in the European Union. Approximately one in 11 women will be diagnosed with breast cancer before the age 74 (Dafni et al., 2019; Ferlay et al., 2021). Such a diagnosis and its treatment profoundly affect not only on the women themselves but also those around them, particularly their partners (Keesing et al., 2016; Zimmermann, 2015).

Paying attention to partners' wellbeing is important, as it contributes to positive psychosocial outcomes for women with breast cancer. Beyond this indirect effect, partners' own wellbeing also deserves recognition (Borstelmann et al., 2015; Cohee et al., 2020; Vesentini et al., 2023; Zimmermann, 2015).

Partners report a lower quality of life compared to partners of women without such diagnosis (Cohee et al., 2020). They frequently face challenges in daily life, including reduced time for work and leisure, and often experience feelings of insecurity and anxiety (Goerling et al., 2020; Woloski-Wruble and Kadmon, 2002). In addition, the sexual relationship is often affected, more than in other cancers, negatively impacting quality of life (Castillo et al., 2022). They often report unfulfilled sexual needs, reduced sexual desire, increased sexual inhibitions, and avoidance of sexual activity (Maleki et al., 2022; Nasiri et al., 2012). These issues are largely linked to cancer treatments, which may cause sexual dysfunctions in women, changes in physical appearance and a negative body-image (Castillo et al., 2022; Kocan and Gursoy, 2016).

A satisfying sexual relationship plays an important role in the overall wellbeing of couples, particularly after cancer treatment. Therefore, addressing the sexual dimension may strengthen the couple's relationship, reduce emotional stress and improve psychosocial adjustment (Di Mattei et al., 2021; Hawkins et al., 2009).

To help partners manage sexual challenges, appropriate professional support and information are needed, yet these are often lacking, even for women themselves (Reese et al., 2017; Stabile et al., 2017). Existing information on sexual health mainly focuses on aspects such as fertility and physical sexual functioning, while relational, emotional and social dimensions are seldomly addressed (Miaja et al., 2017; Reese et al., 2017).

While existing research has largely focused on the wellbeing of women with breast cancer, including aspects of their sexual health, the experiences of their partners remain underexplored. Research in this domain remains particularly scarce and often fails to capture in-depth how partners experience sexuality across the breast cancer trajectory and what professional support they may need in this context. Therefore, this exploratory qualitative study aims to provide in-depth insights into partners' sexual experiences and their perceived needs for professional support across the breast cancer trajectory in Belgium, drawing on a small but information-rich sample, fueling future research and oncology practice.

## Method

### Design

In this exploratory qualitative study, sexuality was approached from a holistic perspective, including emotional and relational dimensions of partners' experiences. In-depth interviews with partners were conducted in Belgium, following a pragmatist paradigm, aimed at identifying experiences and practical needs that could potentially be addressed (Creswell and Plano Clark, 2017).

### Population

The research population consisted of adult male partners of women who had experienced breast cancer in Flanders (Belgium), and who were in a relationship before, during and after cancer treatment. This allowed exploration of partners' experiences and needs during the entire illness trajectory. To minimize retrospective recall limitations, the breast cancer diagnosis should not have occurred more than 10 years ago. Couples with pre-existing sexual problems or involvement in relational therapy before the cancer diagnosis were excluded.

### Recruitment

Participants were recruited via the breast clinic of a university hospital (UZ Brussels). Study flyers were distributed during consultation by attending physicians to eligible partners present or to women with eligible partners, with the request to pass the flier on. A poster was displayed in the breast clinic's waiting room. Both materials briefly described the study and showed a QR code linking to an information page with the researcher's contact information. Additionally, recruitment appeals were shared via social media and personal networks, encouraging reposting.

### Data collection

Interviews were conducted between January 2024 and July 2024 by WG. Interested participants contacted the researcher and received an informed consent form. After consent, an interview was scheduled. Interviews took place online via Microsoft Teams or in person at a participant-chosen location and lasted about an hour (ranging from 45 to 75 min). Interviews were audio recorded and transcribed verbatim.

### Measuring instrument

Interview topics were initially determined by a search in scientific literature (Champion et al., 2014; Goerling et al., 2020; Keesing et al., 2016; Maleki et al., 2022; Nasiri et al., 2012; Vesentini et al., 2023; Woloski-Wruble and Kadmon, 2002). Then, the research team iteratively refined and structured these topics during several team discussions. In this process, attention was paid to the sequencing of questions, starting with more general and less sensitive topics before gradually addressing more intimate aspects of sexuality, in order to facilitate rapport and build trust. Open-ended questions and prompts were incorporated to elicit rich, detailed responses. Finally, the interview guide included a closing phase with less sensitive questions and space for participants to reflect on the interview, ensuring a respectful and appropriate conclusion. This process resulted in the final interview guide (Table 1).

**Table 1:** Interview guide.

Topics	Questions
Illness trajectory	Briefly outline your partner's illness trajectory (diagnosis, treatment, …).
Relationship	How evolved your relational functioning (before, during, and after treatment)?
	How affected breast cancer your relationship (negative and/or positive)?
	How did you cope with sexuality as a couple?
Sexuality	How evolved your sexual functioning (before, during, and after treatment)?
	How affected breast cancer sexuality (negative and/or positive)? Which factors were important herein?
	Can you tell me some anecdotes, painful, or moving memorable moments?
	How was communication with your partner about sexuality? Which factors made it easier or difficult?
	What are your psychological or emotional needs, which could improve sexual functioning?
	What are your social needs (e.g., support from family, friends, peers)
Breast clinic	What was the role/involvement of the breast clinic regarding sexuality?
	Was the topic of sexuality discussed?
	Was information given about sexuality?
	Where there other supportive measures?
	What are your recommendations and suggestions?

### Analyses

Interviews were inductively thematically analyzed by WG (Braun and Clarke, 2008), using the software NVivo (QSR International Pty Ltd., 2018). After transcribing the audiotapes, data were repeatedly reviewed to develop initial coding ideas (open coding). A detailed line-by-line analysis refined these into specific themes and subthemes through code comparisons (axial coding). Finally, core themes were identified and integrated into a narrative describing the results (selective coding). To enhance reliability, all transcripts were also independently read by LV and throughout the iterative process WG and LV continuously reflected and discussed codes, themes and the emerging narrative. Discrepancies were explored and resolved through discussion leading to refinement of the coding structure and themes. Analysis continued until data saturation was reached.

### Reflexivity statement

The interviewer (WG) is a female researcher with a bachelor's degree in medicine, but no clinical experience. While this background contributed to a basic familiarity with breast cancer, her position as a non-clinician may have facilitated a more open and exploratory approach to participants' experiences. She had no prior relationship with the participants, supporting an open and non-assumptive approach to the interviews. Recognizing that complete objectivity is not possible in qualitative research, reflexivity was actively addressed throughout the study.

To enhance reflexivity and limit potential bias, the interviewer maintained a reflexive journal during data collection and analysis. After each interview, notes were recorded on initial impressions, emerging ideas, and potential assumptions, and sensitivities related to discussing sexuality. These reflections were regularly discussed with the supervisor (LV), supporting critical reflection on the researcher's influence on data interpretation.

WG also conducted the initial coding and developed a preliminary codebook in close collaboration with LV. Data analysis followed an iterative and reflexive process, in which codes and themes were continuously reviewed and refined through team discussions. This process enabled critical dialogue and challenged individual interpretations, thereby enhancing the conformability of the findings. Subsequently, the full research team engaged in discussions to examine the coherence, consistency, and distinctiveness of the emerging themes and subthemes, contributing to the dependability of the analysis. Consensus was reached on the final thematic structure and its representation in a coherent narrative.

The credibility of the findings was supported through in-depth interviews that allowed participants to elaborate on their experiences, as well as the use of verbatim quotes to illustrate the themes and ensure transparency. In addition, the inclusion of participant characteristics provides contextual insight, further strengthening the interpretation of the data.

### Ethics

The qualitative study was approved by the Medical Ethics Committee UZ Brussels—VUB (B.U.N 1432023000277).

Participants were well-briefed and signed an informed consent before the start of the interview, which described in detail the aim and design of the study participants, how data is protected, privacy assured, and that participation is voluntary. Transcriptions were anonymized and audio recordings were deleted after transcription. The informed consent included contact details of support helplines, which was reiterated at the end of each interview.

### AI use

AI tool ChatGPT (version 5.1, OpenAI) was used to assist with language editing.

## Results

In total, four partners of women diagnosed with breast cancer were interviewed (Table 2). Participants indicated that breast cancer substantially affected their daily live. It impacted partners emotionally, with feelings of uncertainty and powerlessness, and led to shifts in role patterns, by taking on additional responsibilities. They struggled with altered physical appearance and attraction of their female partner. Further, there was a disruption and renegotiation of sexual intimacy. They argue for more involvement and recognition of breast cancer's impact on partners and for accessible and responsive professional support for relational and sexual issues, that is also partner-oriented.

**Table 2:** Overview characteristics of participants.

Characteristics	Participants			
	P1	P2	P3	P4
Current age participant	59	35	40	70
Age during diagnosis	56	33	38	61
Age of woman during diagnosis of breast cancer	59	31	36	62
Education	University or college degree	University or college degree	University or college degree	High school diploma
Type of surgery	Mastectomy, reconstruction after 1 year	Mastectomy and reconstruction simultaneously	Breast-conservation	Mastectomy, reconstruction after 2 years
Hormone therapy	Ongoing	Ongoing	Ended in 2023	Ended in 2024
Duration relationship	14	14	21	52

### Experiencing and adapting to change within the partner relationship

#### Living with uncertainty and powerlessness

Breast cancer confronted partners with a profound sense of uncertainty and powerlessness. They described not knowing how to handle the situation or how to be supportive, as stated by P2: “*But as a man, you feel powerless because there's nothing you can do to ease it*.” P1 mentioned: “*I want to take away her pain, but I can't*.”

This uncertainty was particularly evident in relation to prognosis and survival. P3 mentioned: “*You have so many questions and uncertainties. Her tumor was very aggressive. You think about the worst-case scenario. Those first few weeks were hell*.”

To deal with this uncertainty, P3 began an almost obsessive search for information to regain some sense of control, as he mentioned himself: “*They could have hired me in the oncology department. I read everything on the internet. I looked up everything mentioned in the medical report. For me, that was a way to gain control over the situation*” (P3).

#### Taking on additional responsibilities

Furthermore, all partners were consistent in indicating that breast cancer affected their daily routines and practical support they had to provide. Role patterns often shifted, as they took on more responsibilities, such as cooking and, when children were involved, childcare. P3 illustrates it as follows: “*Yes, during the treatment, I took on more responsibilities in terms of household chores and raising the children. You want life to continue as normally as possible for the children, so they don't notice too much*.” Although most partners saw this as matter of course rather than a burden, as P4 stated “*She had a lot of side effect, so I cooked. I didn't do it reluctantly*,” one partner mentioned he found it particularly challenging, which led to decreased energy, impacting his work (P2).

#### Struggling with altered physical appearance and attraction

Partners described that side effects of breast cancer treatment affected woman's attractiveness, especially due to the hair loss and chemotherapy, confronting them with the visible reality of illness, as P3 said: “*Yes, chemotherapy really makes you look ill. Somewhat pale and swollen. The hair loss is very hard too. It's really difficult to see your wife like that*.” Likewise, P1 mentioned: “*She was completely bald and looked so swollen. You really don't look like much at all*.”

Further, the mastectomy intensified this experience, especially for P1. He remembered the first time he was confronted with the amputation. Before leaving the hospital, he was obligated to watch the amputated breast, but due to a lack of context and explanation, he felt overwhelmed. “*We weren't' allowed to go home until I had seen the breast amputation. There was no explanation, no conversation. Not about the impact it might have on our relationship. It was just: ‘Here, look, and now you can go home*. *I didn't think it was okay*.”

In one case, the partner's woman had received breast-conserving surgery, while the others had undergone breast reconstruction. In these latter cases, from the partner's perspective such reconstruction was not considered necessary, although they were pleased with the result, as P4 puts it: “*The breast reconstruction was her decision. I didn't push her, but I'm very happy with it, because it is beautiful*.” Further, partners reflected on the complexity of this decision, particularly when they felt that their female partners' decision was influenced by them. For example, P1 mentioned that his wife wanted to do it for him: “*At first, she didn't want a reconstruction, but I'm glad she did. She did it for me, what I think is wrong. I did understand it, though. But now she's also happy with it*.”

### Disruption and renegotiation of sexual intimacy

#### Loss of sexual activity and desire

All partners mentioned a reduction in sexual activity, especially penetration. P2 said: “*In the beginning we still had sex (vaginal intercourse) weekly, but now it's less. It's very painful for her*” and P3 stated: “*There is still sexual intimacy, but it's different and less frequent*.” Two partners also mentioned a decrease in their sexual desire toward their female partner, which they linked to changes in their female partners' physical appearance. P3 had difficulties with his female partner's baldness: “*The desire to have sex is also reduced, because of her bald head*,” and P1 regarding the mastectomy: “*The breast amputation… it was not nice to look at. Absolutely not exciting. On the contrary*.”

#### Attempting to adapt sexual practices

Partners described various ways of adapting their sexual practices due to this new situation. They mentioned using lubrication or vibrator, having gentler and shorter sexual contacts, as P4 shared: “*She did suffer from vaginal dryness. We got some products for that in the pharmacy. And then it's about being gentle and caring with each other*.” Others, like P3, mentioned using a hat during sexual activity: “*She wore a hat to cover her baldness during sex. You don't want to see your wife with a bald head*.” Vaginal intercourse was also more often replaced by oral sex or manual stimulation. As P2 described: “*We have found other ways to reach orgasm. Vaginal intercourse is the best, but now we use our hands. This variation is okay*.” P3 stated: “*There was still room for sex. Often in a different way, though, using hand or orally*.”

At the same time, these adaptations did not fully compensate for what was perceived as a loss of previous sexual experiences. For example, for P1, adaptation was challenging and accompanied by frustration and a sense of loss: “*You can solve it with lubricant, but it's different than it was before. That's an extra element that comes into your sex life and you have to be able to deal with that. We tried, but all that fuss and stuff... Just leave it. And that's what I miss*.”

#### Navigating in sexual communication

Partners did not discuss sexual issues with family or friends, as they considered this private. Communication about sexuality with their female partner varied between participants. While partners P2 and P4 reported to have a good and open conversations, as reflected in following statements: “*It was not a taboo for us. We communicated openly about it*” (P4) and “*She tells me what she likes and what isn't working. We talk about that a lot anyway*” (P2), others, like P3 rather indirectly communicated, trying to sense what the other wanted: “*When you hug each other, when you move on to more sexual activities, it becomes clear what is possible in this context*.” P1 experienced the greatest communication difficulties. He describes a common situation (i.e., his female partner desperately wants him to have an orgasm, possibly as reassurance that he still loves her, but he feels uncomfortable with it) which he cannot discuss with her: “*It's becoming a huge issue between us, but we cannot talk about it anymore. She thinks that I don't love her anymore because she is amputated, but that's not true*.” Another example he gave: “*I also found her less attractive, but I never dared to tell her that*.”

### Shifting closeness: between strengthened bonds and emerging distance

Throughout the trajectory, the impact on the partnership evolved. Initially partners described increased closeness, as P1 stated: “*I think the love grew at that moment. You doubt how long you have together. Then the love becomes deeper*.” The initial deepening stabilized during and after treatment. Partners reported that their relationship had neither worsened nor improved by breast cancer. As P2 put it: “*The relationship was not affected, but it wasn't improved either*” or P4 stated: “*Our relationship didn't really suffer because of it, but I can't say it had a positive effect either*.”

For P1, where sexual difficulties persisted, a growing sense of distance was described, as he shared: “*We had a good cuddle relationship, but that is less now. The intimacy feels different now. It's crumbling and it's difficult to pick up the thread again*.” This loss of physical intimacy also affected emotional and relational domains, raising concerns about the future of the relationship, as he explains: “*Spiritual intimacy means emotional closeness in your conversations. I wonder whether distance arises in that, because of the physical distance caused by cancer. I think it does, because in the end, that spiritual bond alone isn't strong enough to save the relationship. There's less tolerance toward the other*.”

### Needs and recommendations regarding professional support

#### Experiencing recognition and involvement of the breast clinic

Although all partners described they felt welcome in the breast clinic and all healthcare providers were very friendly, this did not translate into a sense of meaningful involvement in the care trajectory. Interactions were experienced as superficial, being excluded from care processes and attention to their own needs. As P2 mentioned: “*Sometimes I was asked how I was doing, but it felt rather superficial*” and P3 put it: “*I was left to my fate*,” because he was never personally addressed, not by a nurse, nor by a psychologist. P1 emphasized the importance of recognition, to legitimize their own needs within a care context that implicitly prioritizes the patient: “*I understand that it's about the woman, but honestly, it's always about the woman. That is a self-centered thought, I now, but can't I tell my story too? And not just about sexuality, but simply to acknowledge that we, as partners, are struggling as well*.”

To enhance the feeling of recognition small (symbolic) gestures can be helpful, as P1 suggested sending a “goodie bag” addressed personally to the partner: “*Just the fact that it is sent to me personally would mean a lot to me. It means they see us*.”

#### Accessible and responsive professional support by the breast clinic

Some partners mentioned the need for accessible and responsive professional support. P3 needed such support to cope with his pessimistic thoughts and despair in the first weeks after diagnosis: “*Yes, those first weeks I was in panic. I was desperate. At that moment, I could have used professional help*.” He further explained it was not clear to him if such psychological support was available for him, so he never asked for such support, suggesting a lack of proactive communication by healthcare providers.

Beyond acute stress, P1 expressed a need for professional support regarding relational and sexual challenges. Initially he tried to discuss this with the psychologist at the breast clinic but felt he was being brushed aside, reflecting a mismatch between partner's needs and the structure or timing or available support: “*I followed the psychologist at the breast clinic with the request to talk about this. She said there would be an exploratory conversation after the surgery. But I thought…. I'm here now, right? This psychological support is worthless here*.” After this incident, he searched in vain for other professional support.

Furthermore, he (P1) emphasized the need for ongoing mental support throughout the entire illness trajectory, starting with coping with and mourning the loss of the breast, followed by guidance on intimacy and finally addressing sexuality: “*You don't go straight to a professional for sexuality problems. I want to build a relationship of trust to make it discussable. You should be able to integrate something like that into guidance that the breast clinic provides*.” This suggests a preference for integrated, longitudinal guidance, rather than *ad hoc* consultations.

#### Lack of partner-oriented information

All partners indicated that no information was available specifically directed to partners. Regarding sexuality, the information provided was limited to the physical side effects and the recommendation to use condoms to avoid pregnancy, leaving broader relational and emotional dimensions unaddressed.

Partners missed several aspects, although there were differences in what they wanted to know more about. P4 expressed that he would have appreciated more information about the sexual experiences from the woman's perspective, as he said: “*But how a woman feels sexually, that would have been interesting to know. A guide for men about what we should consider, would be useful*.” This points to a broader need for guidance that facilitates empathy and mutual understanding within the couple. Other partners mentioned they wanted more information about alternative form of sexual intimacy when penetration is (temporarily) not possible, as P2 puts it: “*I think it is important that the breast clinic supports couples who have intimacy problems. For more conservative couples, it should be explained that there is much more to sexuality than traditional vaginal intercourse*.” This reflects a need for healthcare providers to actively broaden the conceptualization of sexuality presented to couples. Furthermore, partners also would have appreciated information about the possible use of sexual aids or sex toys, as P3 said: “*There are other options or resources available. It wouldn't hurt to learn more about them*.” Finally, P1 indicated that he needed more information about how the physical side effects could affect the relationship, both in terms of intimacy and sexuality, and emotionally: “*They should have said what a mastectomy can do with you. That it can seriously affect how you, as a man, perceive your wife. That you can find an amputated breast a turn-off. They need to name that, because you can't say such thing to your wife*.” This illustrates how unaddressed concerns might burden the partner, potentially affecting both intimacy and emotional connection.

Receiving written information material such as flyers and brochures was considered important. Not only partners had to search for answers themselves, but they also believed that information could help make delicate issues such as sexuality more open to discussion, both with their female partner and with healthcare providers, as P1 stated: “*That would provide an opportunity to talk about intimacy*.” In addition to written information, a scheduled consult was considered valuable. Partners advocated for consultations or group sessions specifically designed for men, like P4: “*I think it would be interesting if there was a session just for men*.” This suggestion aligns with P1's need for testimonies from fellow sufferers about the emotions they struggle with.

Finally, all partners agreed that providing information about sexuality should be systematically integrated in the provided care. There was, however, no consensus on the optimal timing for providing this information. One partner stated that it was unnecessary at the start of the trajectory and that timing should be determined by a psychologist, whereas others felt it should be addressed as early as possible, as P4 stated: “*The earlier you understand how things develop and how it works, the better. The information can be future-oriented, but at least then you're prepared*.”

## Discussion

Findings of this exploratory qualitative interview study revealed that breast cancer diagnosis and treatment seem to leave partners feeling uncertain and powerless, especially in the beginning. It also led to role shifts, with them taking on more household responsibilities. Changes in the female partner's appearance affected the interviewed partners and influenced sexuality. Although these partners tried to adapt, communication about sexuality was not self-evident for everyone, which appeared to affect the relationship. All partners felt their needs were underrecognized by healthcare providers and reported a lack of professional support on mental, sexual and relational levels. They reported that information on these topics was largely absent, despite its potential to facilitate conversations with their wife and healthcare providers. According to them, such information should be offered systematically, for example through flyers or during consultations. Finally, partners expressed a need for consultations or group sessions solely for male partners.

A key strength of this study is its focus on an underexplored topic, contributing to more insight into the sexual challenges and professional support needs of partners of women diagnosed with breast cancer. Although the small number of participants is a limitation, the interviews yielded rich data, particularly from partners experiencing difficulties within their (sexual) relationship. However, the limited sample size may have restricted the diversity of perspectives and the identification of variation across experiences. The findings should therefore be interpreted with caution and are not intended to be transferable, but rather to provide in-depth insight into this underexplored phenomenon. In addition, although efforts were made to minimize recall bias by including only participants whose partners were diagnosed within the last 10 years, this timeframe remains relatively broad. Participants reflecting on experiences from several years ago may have reinterpreted or reconstructed these experiences over time, potentially influencing their accounts. At the same time, this retrospective perspective may also have allowed for reflection on the longer-term impact of breast cancer on sexuality and support needs. Building on these limitations, future research should therefore include a broader and more diverse sample, including former partners, as they are likely to provide the richest insight. Furthermore, largescale quantitative studies are needed to determine the magnitude of these needs.

A main finding was a decrease in sexual activity and desire toward the female partner, related to the cancer treatment, which aligns with previous research (Maleki et al., 2022; Woloski-Wruble and Kadmon, 2002). Sexuality was particularly affected by changes in women's appearance, such as baldness and mastectomy, and by the fact that sexual intercourse no longer felt as natural as before (for some men), which may reflect disruptions in established sexual scripts and expectations surrounding intimacy and the female body (Gilbert et al., 2010; Loaring et al., 2015).

Partners tried to adapt to this new situation by concealing baldness, exploring alternative sexual practices, and using lubrication and sex toys. These adaptive strategies might suggest active efforts to renegotiate intimacy within constraints of illness (Gilbert et al., 2010; Loaring et al., 2015). Nevertheless, they emphasized the need to provide information as a standard procedure on these issues, including how women experience these sexual changes. Besides information, partners desired professional support, such as guidance in coping with the loss of a breast and preparing for the relational impact of physical changes. However, such support was never received, which is consistent with findings of (Albers et al. 2020), where 73.7% of partners reported receiving no information regarding sexuality. Beyond sexual related support, partners needed also emotional support and general information about breast cancer, particularly early in the trajectory, as information seeking can serve as a coping strategy (Keesing et al., 2016; Woloski-Wruble and Kadmon, 2002). Together, these findings highlight a persistent gap in partner-oriented care, particularly regarding sexuality, which remains insufficiently addressed in clinical practice.

Due to the lack of partner-focused guidance, participants felt underrecognized and left to their fate, which may be understood in light of caregiving role expectations, where partners prioritize the patient's needs over their own (Chin et al., 2025; Goerling et al., 2020). They wished for ongoing professional support throughout the entire cancer trajectory, preferably on their own. This is an important finding, as it nuances dyadic coping approaches, which emphasize shared coping and mutual support and are associated with higher relationship satisfaction (Badr and Krebs, 2013; Zimmermann, 2015). While dyadic models assume openness and mutual exchange, partner-only interventions may be beneficial, as male partners often refrain from expressing their needs to avoid burdening their female partners (Cheng et al., 2014; Kleine et al., 2019). These findings therefore suggest that, alongside dyadic approaches, individual support formats may better accommodate partners' tendency toward protective buffering, whereby they downplay or withhold their own needs (Goerling et al., 2020; Manne et al., 2007).

Finally, literature indicates that breast cancer negatively affects the relationship in 25% of partners, mainly due to communication difficulties, and leads to the end of relationships in 12% (Walsh et al., 2005). This was also reflected in this study, where relationships tended to remain stable or deteriorate over time. One partner who struggled most with adaptation and communication considered ending the relationship. Although based on a small sample, this finding provides a nuanced illustration of how relational strain may emerge when communication and adaptation processes are challenged (Gilbert et al., 2010). This is concerning, as open communication might help couples maintain (sexual) intimacy, adjust expectations, and manage changes together (Loaring et al., 2015; Rowland and Metcalfe, 2014).

## Conclusion and implications for practice

Overall, this exploratory study extends existing research by offering preliminary, in-depth insights into partners' sexual experiences across the breast cancer trajectory, drawing attention to their often-overlooked perspectives. It highlights the potential added value of individual, partner-focused support alongside dyadic approaches and identifies specific informational and supportive care gaps that remain insufficiently addressed in current practice. Therefore, it may be helpful to consider providing partners with a personally addressed box containing tailored information. While these findings and recommendations should be interpreted with caution, they point to persisting informational and supportive care gaps that warrant further investigation.

## Data Availability

The datasets presented in this article are not readily available because participants did not gave written consent for their data to be shared publicly, due to the sensitive nature of the research. Requests to access the datasets should be directed to lara.vesentini@vub.be.
